# Role of Histone Lactylation in Neurological Disorders

**DOI:** 10.3390/ijms26167949

**Published:** 2025-08-18

**Authors:** Yu-Mo Zhang, Fei Yang, Qian Li, Jian-Nan Zhang

**Affiliations:** 1 School of Basic Medical Sciences, Capital Medical University, Beijing 100069, China; 2 Department of Neurobiology, School of Basic Medical Sciences, Capital Medical University, Beijing 100069, China; feiyang@ccmu.edu.cn; 3 Laboratory for Clinical Medicine, Capital Medical University, Beijing 100069, China; 4 Department of Biochemistry and Molecular Biology, School of Basic Medical Sciences, Capital Medical University, Beijing 100069, China

**Keywords:** histone lactylation, Alzheimer’s disease, depression, neuroinflammation, aging

## Abstract

Lactate is not only the end product of glycolysis but also plays a key role in epigenetic regulation. Recently, lactate-derived histone lactylation has been identified as a novel epigenetic modification that can directly influence gene transcription. Histone lactylation has been associated with various pathological conditions and shows significant therapeutic potential. However, studies on histone lactylation in central nervous system diseases are still quite limited. In this review, we summarize the latest research progress on histone lactylation, detailing the specific mechanisms and sites of histone lactylation, including lactylation and delactylation. We also discuss the role of histone lactylation in Alzheimer’s disease (glycolysis/H4K12la/PKM2 feedback loop), depression (neuronal excitation), neuroinflammation (anti-inflammatory/pro-inflammatory balance of microglia), aging, stroke (infarct volume), and glioblastoma (activation of oncogenes), pointing out the research directions for the future. This may provide new ideas for the diagnosis and treatment of neurological diseases.

## 1. Lactate and Lactylation

Lactic acid is the end product of cells undergoing anaerobic glycolysis, and its production regenerates the NAD^+^ consumed during the process of glycolysis [1]. L-lactic acid, a metabolic intermediate that can be converted through gluconeogenesis, is transported between different cells and organs in the body. It plays an important role in many physiological processes. Under pathological conditions such as hypoxia or certain cancers, cells may produce L-lactic acid at higher rates. In hypoxic conditions, cells switch to anaerobic metabolism, which leads to increased L-lactic acid production [2]. Similarly, cancer cells often exhibit heightened glycolytic activity even in the presence of adequate oxygen (the Warburg effect), resulting in increased L-lactic acid production as a metabolic adaptation [3]. Moreover, L-lactic acid is also a vital signaling molecule in the brain, and elevated levels of lactate have been observed in many neurological disorders [4].

Recent research has found that lactic acid has a new role in the field of epigenetic research. Epigenetics refers to changes in gene function that can be inherited without changing the DNA sequence, ultimately leading to changes in phenotype. Post-translational histone modifications comprise part of the hierarchy of epigenetic regulatory mechanisms [5]. Various histone acylation marks derived from cellular metabolites have been discovered in recent years, such as propionylation, butyrylation, 2-hydroxyisobutyrylation, succinylation, malonylation [6], and so on. These histone modifications alter the spatial structure of proteins, regulating many physiological and biochemical processes in cells [7]. Histone lactylation, derived from lactate, was recently identified as a novel histone modification [8].

Lysine lactylation (Kla) is a post-translational modification that is stimulated and regulated by lactic acid [8]. There are three isomers of Kla: L-lactyl-lysine (K_L-la_), N-ε-(carboxyethyl)-lysine (K_ce_), and D-lactyl-lysine (K_D-la_). It has been proven that neither K_ce_ nor K_D-la_ are present on histones in wild-type cells [9]. Meanwhile, K_L-la_ is susceptible to glycolysis. The initial evidence of histone Kla comes from the discovery of three protein hydrolysis peptides with a mass shift of 72.021 daltons on the lysine residue when analyzing core histones digested with trypsin in MCF-7 cells using high-performance liquid chromatography (HPLC)–tandem mass spectrometry (MS/MS) [8]. This mass shift is identical to that caused by the addition of a lactate group to the ε-amino group of the lysine residue [8]. Under physiological conditions, lactylation fine-tunes neuroplasticity genes (e.g., Egr1, c-Fos) in response to synaptic activity. Baseline H3K18la levels in hippocampal neurons modulate spatial memory consolidation, while exercise-induced lactylation promotes neurogenesis [10].

Lactic acid, as a precursor, is converted to lactic acid coenzyme A and transferred to histone under the action of acetyltransferase [11]. In M1 macrophages, lactate is a signaling molecule that stimulates gene transcription through histone Kla [8]. Zhang and others have shown that the regulation of intracellular lactate production leads to histone Kla levels changing in a dose-dependent manner. In addition, intracellular metabolic disorders are associated with the functionality of epigenetic writers and erasers, while epigenetic writers and erasers can also regulate histone acetylation and lactylation [12]. Critically, lactate shuttling between astrocytes and neurons regulates synaptic plasticity via lactylation-dependent gene expression such as BDNF [13]. Dysregulated lactylation in neurological contexts may thus disrupt neurometabolic coupling, exacerbating disease progression.

While prior reviews have summarized histone lactylation in cancer and immunology, this is the first comprehensive synthesis focusing on its mechanistic roles in neurological disorders (AD, depression, neuroinflammation, aging), highlighting novel feedback loops (e.g., PKM2/H4K12la in AD) and therapeutic implications specific to the central nervous system (CNS).

## 2. Addition and Removal of Lactyl Groups

### 2.1. Lactylation: Enzymatic and Non-Enzymatic Reactions

**Figure 1:**
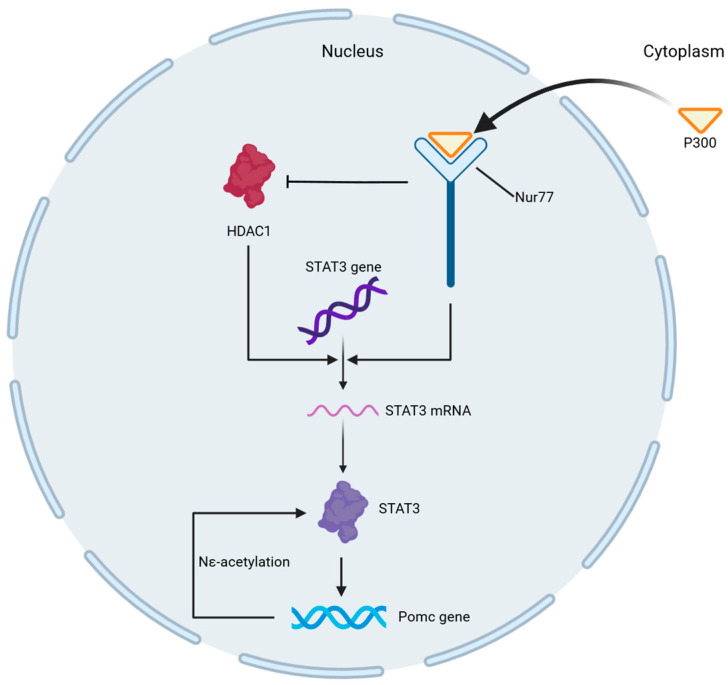
**Regulation of histone lactylation enzymatic reactions through nuclear receptor pathways.** The nuclear receptor Nur77 recruits P300, reduces HDAC1 expression, and enhances the transcriptional activity of STAT3 through N-ε acetylation. P300, a key lactyltransferase, is recruited here for acetylation; its role in lactylation may operate via analogous mechanisms. Nur77: nuclear receptor 77; P300: lysine acetyltransferase enzymatic protein 300; STAT3: signal transducer and activator of transcription 3.

Lysine acetyltransferase (KAT) enzymatic, P300, and lactyl-CoA jointly result in enzyme-catalyzed Kla [8]. It has also been found that the nuclear receptor Nur77 recruits P300, reduces HDAC1 (histone deacetylase 1) expression, enhances the transcriptional activity of signal transducer and activator of transcription 3 (STAT3) through Nε-acetylation, and regulates the expression of the downstream gene *Pomc* in the hypothalamus, thereby creating a positive feedback loop that promotes the expression of STAT3. Both pathways mentioned above jointly regulate histone lactylation and form the enzymatic parts [14]. Although Nur77-P300-STAT3 primarily regulates acetylation, this pathway exemplifies how acetyltransferases like P300 can be recruited for lactylation, given P300′s dual role in both modifications (Figure 1) [11]. Zhu et al. recently reported on lactyl-CoA synthetase and lactyltransferase as well as their role in histone lactylation. They found acetyl-CoA synthetase 2 (ACSS2) functions as a lactyl-CoA synthetase and converts lactate to lactyl-CoA [15]. Lactyl-CoA is subsequently transferred to histones by KAT, primarily P300 and KAT2A [8,15]. Specifically, KAT2A catalyzes site-specific lactylation at H3K14 and H3K18, while P300 may function as a complementary lactyltransferase recruited by nuclear receptors (e.g., Nur77) [15]. In addition to the enzyme-dependent transfer of lactyl-CoA to lysine residues, lysine acylation has been shown to originate from a non-enzyme reaction with lactic glutathione (LGSH) [11,16]. Methylglyoxal is a byproduct of sugar metabolism that reacts enzymatically with glutathione to form LGSH. LGSH is then hydrolyzed by GLO2 to produce glutathione and D-lactate. Gaffney and others recently discovered that non-enzymatic lactate transfer from LGSH to protein lysine residues can lead to “LactylLys” modification in the protein, which can be inhibited by GLO2, thereby regulating glycolysis [16]. In addition, in mitochondrial proteins, the non-enzyme-promoted lysine acetylation of acetyl-CoA and acetylglutathione is promoted by proximal S-acetylation thiol intermediates, which can be restrained by GLO2 [17].

### 2.2. Delactylation

Mammals express two families of lysine deacetylases, comprising a total of 18 enzymes. The histone deacetylases (HDAC1-11) rely on Zn^2+^ [18], while sirtuins (SIRT1-7) depend on NAD+ as a cosubstrate [19]. Histone delactylation is dynamically regulated by class I histone deacetylases (HDAC1-3), which exhibit specific lysine delactylase activity [20,21]. Among these, HDAC1 and HDAC3 are the primary delactylases that target lactylation sites such as H4K5la and H3K18la [20]. This functional specificity is attributed to their integration into multi-subunit complexes that confer substrate selectivity [22]. These complexes contain scaffold proteins that interact with other epigenetic enzymes, such as demethylases, as well as with transcription factors [22]. When HDACs bind to their respective complexes, their activity is enhanced [23]. HDAC1-3 show different substrate selectivity in cells, possibly due to interactions with different complex partners [24]. It has also been found that HDAC1 and HDAC3 can significantly change the lactylation level of the H4K5 site, while HDAC2 cannot. The study suggests that the impact of each HDAC isoform on overall histone lactylation levels is limited. HDAC1/2 are often present in the same multi-protein complex in the cell nucleus and are believed to compensate for each other in the regulation of histone Kla [22]. HDAC1/3-mediated delactylation demonstrates that histone Kla is dynamically regulated, akin to acetylation, offering new targets for epigenetic therapeutics. These enzymes in the cell nucleus delactylate histones, thereby achieving reversible and dynamic regulation of histone lactylation [25]. This result, along with the chromatin immunoprecipitation sequencing observation of histone lactylation, strongly demonstrates the potential genome-wide localization specificity of histone lactylation [8], providing powerful evidence that histone l-lactylation may be a major pathway for nuclear lactylation [20]. In summary, these findings not only provide new insights into the novel activity of Class I HDACs, but also validate the pharmacologically targetable enzyme regulation of histone K, laying the foundation for future functional studies of this modification and its related regulatory pathways [20].

## 3. Pathology Manifestations

Histone lactylation plays an important regulatory role under normal physiological conditions as well as in pathological conditions (such as Alzheimer’s disease (AD) and depression [26]).

### 3.1. AD

AD is a debilitating neurodegenerative condition characterized by a progressive decline in cognitive function [27]. It manifests through impairments in memory, cognition, and behavior. The etiology of AD is mainly attributed to the accumulation of Amyloid-β(Aβ)plaques and tau neurofibrils within the brain parenchyma. These pathological aggregates disrupt neuronal integrity, leading to the characteristic symptoms associated with the disease [28,29]. AD is also a chronic neuroinflammatory disease, accompanied by the abnormal activation of microglial cells, which progressively leads to the dysfunction of microglial cells [30]. Microglia are the resident immune cells of the central nervous system, providing immune protection under pathological conditions [13].

**Figure 2:**
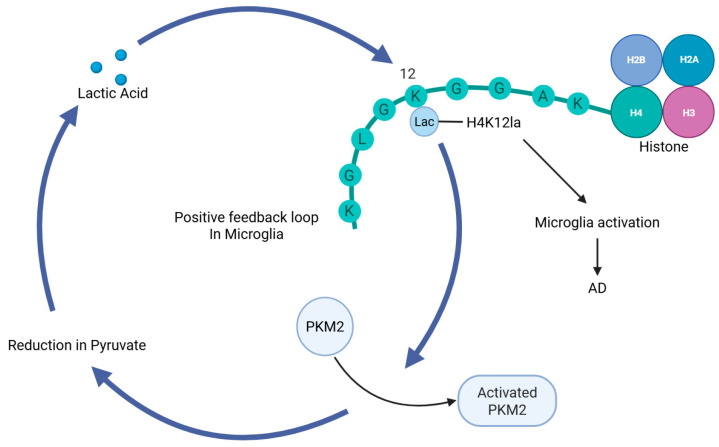
**The positive feedback loop in microglia leads to AD by promoting histone H4K12la.** Upon activation of PKM2, a key enzyme in glycolysis, it promotes anaerobic respiration to produce pyruvate, which is subsequently reduced to lactate. Once lactate enters the nervous system, it induces histone H4K12 lactylation in microglia, leading to the activation of microglia. Lactylation activates PKM2, creating a positive feedback loop that amplifies lactylation, ultimately leading to AD. PKM2: Pyruvate kinase isozyme type M2; AD: Alzheimer’s disease.

In AD, several histone Kla sites, including H4K8, H4K12, H3K14, and H3K18 sites, have been identified in both microglia and neurons in the hippocampus [31]. It has been found that histone lactylation increases in the amyloid β-plaque-associated microglia of AD patients, particularly H4K12la and H3K18la [31,32]. In AD, histone lactylation activates the transcription of glycolysis genes, forming a positive feedback loop that further exacerbates microglial cell activation and functional impairment (Figure 2). This loop involves active glycolysis, H4K12la, and pyruvate kinase M2 (PKM2). Further study has also found that inhibition of PKM2 disrupts the glycolysis/H4K12la/PKM2 positive feedback loop in microglia, which can alleviate microglial activation and neuroinflammatory responses. 5XFAD mice represent a commonly utilized genetic model for AD. They are generated through transgenic technology, which involves the introduction of human amyloid precursor protein (APP) and presenilin 1 (PSEN1) mutant genes into the mouse genome [33]. These mutant genes cause the mice to produce large amounts of Aβ plaques, which are one of the typical pathological features of AD. In 5XFAD mice, specific deletion of the Pkm2 gene in microglial cells can reduce β-amyloid deposition and improve spatial learning and memory [32]. Thus, inhibiting the glycolysis/H4K12la/PKM2 feedback loop may be considered a potential strategy for the treatment of AD. Similarly, research also suggests that a positive feedback loop exists for H3K18la. H3K18la enhances NFκB signaling, and facilitates the senescence-associated secretory phenotype (SASP) components IL-6 and IL-8, thereby promoting AD progression through inflammatory signaling pathways [31].

### 3.2. Depression

Depression can lead to a decrease in the number and density of astrocytes in the prefrontal cortex (PFC) of patients, suggesting that astrocyte dysfunction plays a role in the pathogenesis of depression [34]. There is evidence that suggests that fluoxetine and paroxetine promote lactate release from cortical astrocytes [35] and that peripheral administration of lactate can produce antidepressant-like effects, such as reduction in behavioral despair, anhedonia-like behaviors, and social avoidance [36,37]. Chronic corticosterone administration can induce a depressive-like state in mice [38], and peripheral lactate treatment can reverse this effect of corticosterone [36]. In a corticosterone model of depression, antidepressant effects of lactate require adult hippocampal neurogenesis, using the anti-mitotic drug Temozolomide, which impairs neurogenesis in the hippocampus [39]. Corticosterone exerts an augmentative effect on neuronal oxidative stress [40,41,42]. Hippocampal stem cells that have been treated with corticosterone exhibit diminished cell proliferation and an elevated production of reactive oxygen species (ROS). However, co-administration of corticosterone and lactate mitigates these detrimental effects. The above results suggest that the conversion of lactate to pyruvate, accompanied by the generation of NADH, is essential for the neuroprotective properties of lactate in depression. These effects are partially mediated by corticosterone-induced inhibition of ROS [39]. Both lactate and pyruvate enter the central nervous system via monocarboxylate transporter 1 (MCT1) across the blood–brain barrier, but chronic administration of pyruvate failed to produce antidepressant-like effects, in sharp contrast to lactate [43]. This suggests that NADH generated by lactate dehydrogenase during lactate is oxidized to pyruvate may be involved in the neurogenic and antidepressant effects of lactate [39].

**Figure 3:**
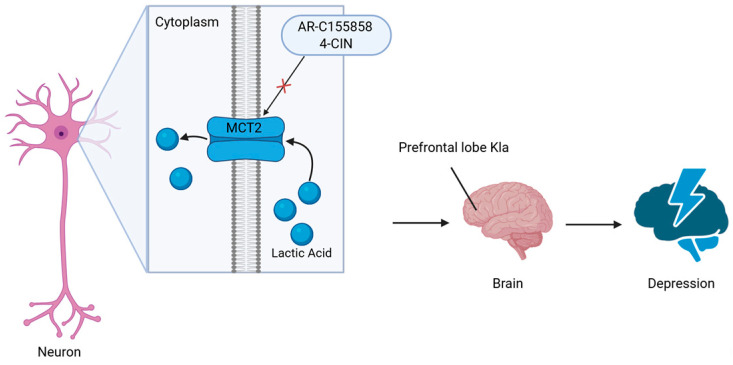
**Lactate transport into prefrontal cortex neurons promotes depression-associated protein lactylation.** Lactic acid molecules produced in the body are transported into prefrontal cortex neurons by MCT2, contributing to Kla, which is associated with depressive symptoms. The inhibitor of MCT2, 4-CIN, dose-dependently attenuated the lactate-induced increase in Kla immunoreactivity, blocking the transport of lactate into neurons, indicating that MCT2 may be involved in the effect of lactate on Kla. The selective MCT1/2 inhibitor AR-C155858 also inhibited the lactate-induced increase in Kla, further supporting the role of MCT1/2 in lactate regulation of Kla. MCT2: monocarboxylate transporter 2; 4-CIN: α-cyano-4-hydroxycinnamate; AR-C155858: C_21_H_27_N_5_O_5_S, the inhibitor of MCT1/2.

Stress, especially chronic stress, has been shown to alter neural activity in the brain [44,45]. To explore the mechanism of brain lactate regulation in depression, the researchers applied an in vitro neuronal excitation model with high-K. They found that the levels of lactate have a time-dependent increase after high-K treatment, and a high-K-induced increase in Kla levels was inhibited by sodium oxamate (OX), which inhibits lactate dehydrogenase (LDH) activity. LDH can inhibit the production of lactate in the central nervous system [46,47]. Thus, neuronal excitation may induce Kla via intracellular metabolism and the glycolytic pathway. The inhibitor of monocarboxylate transporter 2 (MCT2), α-cyano-4-hydroxycinnamate (4-CIN), dose-dependently attenuated the lactate-induced increase in Kla immunoreactivity, blocking the transport of lactate into neurons, indicating that MCT2 may also be involved in the effect of lactate on Kla [48]. Further study in mice with electroconvulsive stimulation (ECS) in vivo found that both intracellular lactate production and extracellular lactate uptake are involved in acute neuronal activation in vivo [48]. C-Fos is an immediate early gene product used as a marker for neuronal activation [49]. Double-immunostaining analysis of c-Fos showed a significant positive correlation between Kla and c-Fos immunoreactive intensities, suggesting that more Kla is induced in the cell when more neurons are activated [48]. Finally, they examined whether Kla was affected by behaviorally induced neuronal excitation associated with SDS. The results showed that Kla levels in the PFC were associated with anxiety-like behaviors, suggesting a potential behavioral significance of Kla in the brain. In addition, they highlighted that histone H1 lactylation, out of 63 candidate lactylated proteins, increases in the mouse PFC in response to chronic stress. Overall, this study provides evidence for the role of neuronal activity-induced lactate mediated by protein lactylation (Figure 3) [48].

### 3.3. Neuroinflammation and Aging

Neuroinflammation is a common pathological feature of many acute and chronic neurological diseases. It exerts a devastating impact on cells and brain function and accelerates the progression of long-term neurodegenerative diseases [50]. Neuroinflammation may be a cause or a consequence of disease progression at different stages of the disease. Inflammation can cause damage to cells and brain function by suppressing neurogenesis and accelerating neurodegeneration [51].

To investigate the role of lactate-induced Kla in promoting a reparative microglial phenotype in neuroinflammation, researchers incubated the BV2 microglial cell line with Aβ1-42 to induce activation and examined the mRNA expression of pro-inflammatory gene IL-1β and reparative genes Arg 1 and VEGF [10]. The results revealed that the IL-1β gene was sharply upregulated at the initial stage of Aβ 1-42 inflammatory stimulation, and then gradually declined over time, indicating that exogenous lactate treatment promotes the transformation of pro-inflammatory microglia to anti-inflammatory/reparative phenotype by lactylation [10].

**Figure 4:**
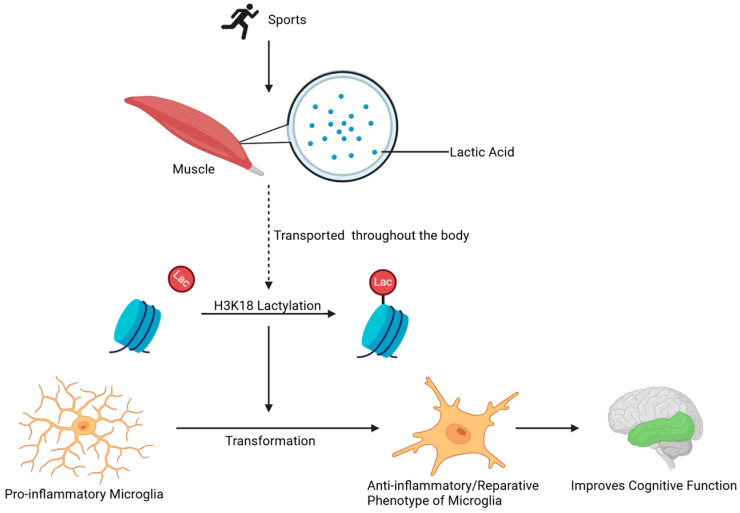
Lactic acid produced during exercise is transported from muscle cells into microglia, promoting their phenotype transformation through histone lactylation. During exercise, lactic acid is produced in muscle cell cytoplasm through anaerobic respiration and amplifies microglial transition from a detrimental to a reparative phenotype via H3K18la lactylation of microglial histone H3.

In addition to in vitro experiments, lactate molecules mediated by anaerobic respiration during exercise also play a crucial role in neuroinflammation in vivo. Lactic acid is a major product of exercise and is transported by the blood to various tissues throughout the body [52]. Also, lactate levels rise in muscle cells after running exercise [53]. Lactic acid produced by exercise amplifies microglial transition from a detrimental to a reparative phenotype via H3K18 lactylation of microglial histone, which improves neural and cognitive function in a mouse model. Lactylation enrichment and increased arginase 1 expression in macrophages was observed when these cells were treated with exogenous lactate [8,53]. In this process, lactate plays the role of a “timer” in the transition of macrophages from a pro-inflammatory phenotype to an anti-inflammatory/reparative phenotype [10]. Finally, the researchers demonstrated that the addition of lactate accelerated the same transition of the detrimental to the reparative phenotype as cultured BV2 microglia. Thus, exercise training or lactate administration may act as an “accelerator” for the microglial intrinsic “lactate timer”, with histone lactylation improving cognitive function via modulating the anti-inflammatory/pro-inflammatory balance of microglia in conditions such as neuroinflammation and aging (Figure 4) [10,54]. Researchers demonstrated that exercise-induced lactate elevation in the mouse brain also acts as an endogenous accelerator of the “lactate timer” in microglia in various aged mouse models induced by D-gal and AlCl_3_ [10].

Aging is a risk factor for neurodegenerative diseases [55]. D-galactose (D-gal) is an aging agent, while aluminum is a known neurotoxin associated with the onset of AD. There is abundant evidence that D-gal/aluminum chloride (AlCl_3_) combined treatment can induce AD-like symptoms, including cognitive and memory impairment, amyloid-β overexpression, oxidative damage, microglia activation, and neuroinflammation [56,57].

### 3.4. Histone Lactylation in Other Neurological Disorders

Stroke is one of the leading causes of mortality and disability globally, presenting substantial burdens to society and healthcare systems [58]. Among stroke subtypes, ischemic stroke constitutes the most prevalent form [59]. Evidence indicates that lactate production is closely linked to the progression of ischemic stroke [60]. In ischemic stroke, lactate accumulation during reperfusion injury exacerbates blood–brain barrier disruption and neuronal death. A recent study revealed that the knockdown of the lactate dehydrogenase A (LDHA) protein could reduce histone lactylation levels at the HMGB1 promoter, as well as decrease the expression levels of IL-18, IL-1β, cleaved caspase-1, and GSDMD-N proteins in both in vivo and in vitro models of cerebral ischemia/reperfusion. This intervention was associated with a reduction in infarct volume and an improvement in neurological function in MCAO rats [61]. In addition, lactate-driven histone lactylation (particularly H3K9la and H3K18la) dysregulates gene expression in the neurovascular unit, amplifying pro-inflammatory pathways and impairing tissue repair [62].

Glioblastoma is the most common and aggressive primary brain tumor in adults [63]. For glioblastoma, the Warburg effect creates a hyperlactate microenvironment that epigenetically fuels tumor progression [64]. H3K9la directly activates oncogenes by recruiting bromodomain-containing protein 4 (BRD4) to enhancer regions, driving glioblastoma cell proliferation and therapy resistance [26,65,66]. As a lactylation “reader”, BRD4 recognizes H3K9la/H4K8la marks and recruits transcriptional machinery to activate oncogenes [26,66]. In subarachnoid hemorrhage (SAH) models, BRD4-mediated binding to H4K8la drives astrocyte pro-inflammatory polarization [66]. The study confirmed that BRD4 mediates A1 polarization of astrocytes after SAH by regulating histone lactylation, particularly H4K8la. Knockdown of BRD4 significantly reduced histone lactylation levels but did not affect glycolysis or lactate production. Experiments confirmed that BRD4 directly binds to H4K8la. Under OxyHb stimulation, the absence of BRD4 inhibited the upregulation of H4K8la and the expression of the pro-inflammatory marker C3, leading astrocytes to secrete more IL-1β, TNF-α, and IL-6. These inflammatory factors further reduced neuronal viability and exacerbated neuronal death [66]. These findings collectively highlight histone lactylation as a critical metabolic–epigenetic nexus across diverse neurological disorders, offering novel targets for therapeutic intervention.

### 3.5. Cell-Type-Specific Regulation of Histone Lactylation in the CNS

Emerging evidence reveals that histone lactylation exerts cell-type-dependent functions across neural populations, with distinct pathological and therapeutic implications (Table 1).

In Aβ-activated microglia, exogenous lactate promotes H3K18la-mediated upregulation of reparative genes (Arg1, VEGF), facilitating phenotype switching via a “lactate timer” mechanism [10]. AD-associated microglia exhibit elevated H4K12la/H3K18la, driving *PKM2*-dependent glycolytic feedback loops that exacerbate neuroinflammation [31,32]. Exercise-induced lactate further amplifies H3K18la-enriched anti-inflammatory microglial states, improving cognition in aging models [10,54]. Astrocyte–neuron lactate transport regulates synaptic plasticity through BDNF lactylation, essential for long-term memory formation [13]. Cortical astrocytes release lactate under fluoxetine/paroxetine treatment, while chronic stress reduces astrocyte density in the PFC—suggesting that astrocytic lactate dysregulation is a core component of depression etiology [34,35]. Neuronal excitation (high-K^+^ stimulation or electroconvulsive shock) induces time-dependent Kla, particularly on histone H1, correlating with c-Fos activation markers [48]. MCT2-mediated lactate uptake in prefrontal neurons promotes Kla modifications linked to anxiety-like behaviors, whereas corticosterone-induced oxidative stress is mitigated by lactate-derived NADH generation [39,48]. Cell-specific lactylation signatures may enable precision targeting of neuropsychiatric disorders. Future studies should leverage single-cell epigenomics to resolve spatial heterogeneity in lactylation landscapes [67].

## 4. Drug Development

Based on the core regulatory mechanisms of histone lactylation in neurological disorders, future drug development should focus on synergistic interventions targeting pathological feedback loops, lactylation enzyme systems, and lactate transport pathways [8]. For the critical link of aberrant microglial activation in AD, highly selective small-molecule PKM2 inhibitors can be developed to disrupt the “glycolysis/H4K12la/PKM2” positive feedback loop, while designing HDAC1/3-specific cyclic peptide inhibitors to block the neuroinflammatory cascade mediated by the H3K18la/NFκB pathway [31,68,69]. At the enzymatic regulation level, allosteric modulators of ACSS2 (lactyl-CoA synthetase) should be constructed to precisely control lactyl-CoA biosynthesis, and competitive KAT2A inhibitors should be developed to block pathological lactylation modifications at H3K14/H3K18 [20,70]. For lactate metabolism and transport, optimization of blood–brain barrier-penetrant MCT1/2 dual-function modulators is required to achieve brain region-specific lactate flux regulation, alongside designing “exercise-mimetic compounds” to directly elevate brain lactate concentration and promote H3K18la-dependent reparative microglial phenotype transformation [71]. Additionally, integrated diagnostic-therapeutic strategies should be implemented, developing novel PET tracers based on H4K12la/H3K18la epigenetic maps to provide dynamic monitoring for disease stratification and efficacy evaluation [67]. This R&D framework necessitates combining cryo-electron microscopy to resolve lactyl–CoA–histone complex structures for rational drug design, utilizing single-cell epigenomics to screen cell-type-specific targets, and employing nanocarrier delivery systems to overcome blood–brain barrier limitations. Ultimately, validation in models will demonstrate translational value, driving breakthroughs in histone lactylation regulation, from mechanistic research to clinical therapeutics.

## 5. Limitations and Future Perspectives

Histone lactylation represents a druggable node in neurological diseases, such as suppressing H4K12la via PKM2 inhibitors ameliorates AD pathology, while exercise mimetics that boost H3K18la may combat depression. Precision targeting of lactylation dynamics could yield disease-modifying therapies. The burgeoning field of histone lactylation has unveiled its critical roles in neurological disorders, yet numerous questions remain unanswered. Future research should prioritize the following directions to bridge existing gaps and translate findings into clinical applications.

Firstly, there is a critical scarcity of cell-type-specific lactylation data within the human brain. This gap impedes the precise delineation of lactylation’s physiological roles and pathological contributions across distinct neuronal and glial populations. Secondly, the crosstalk and potential regulatory interplay between lactylation and other major post-translational modifications (PTMs), such as acetylation, remains poorly defined. This lack of clarity regarding competitive, cooperative, or hierarchical relationships complicates the interpretation of lactylation’s functional consequences within broader signaling networks. Furthermore, a major translational barrier exists due to the absence of lactylation-specific positron emission tomography (PET) tracers. The lack of such non-invasive imaging tools severely restricts the ability to monitor lactylation dynamics in vivo, assess their relevance regarding disease progression, or evaluate the efficacy of lactylation-targeting therapies in clinical settings. Addressing these limitations is essential for advancing the field.

Secondly, while enzymatic and non-enzymatic pathways for lactylation have been identified, their spatiotemporal regulation across diverse cell types and disease states remains poorly understood. Advanced techniques, such as single-cell multi-omics and cryo-electron microscopy, could resolve the structural basis of lactyl-CoA binding to histones and delineate isoform-specific functions of lactylation in neurological diseases.

Thirdly, the role of histone lactylation in Parkinson’s disease, stroke, and glioblastoma warrants exploration. Furthermore, the glycolysis/H4K12la/PKM2 and H3K18la/NFκB feedback loops in microglia represent promising therapeutic nodes. Developing highly specific HDAC1/3 inhibitors or lactate-CoA synthase modulators can effectively intervene in the pathological lactylation modification cycle. Concurrently, exercise-mimetic compounds that enhance lactate flux without physical exertion might offer novel strategies to amplify reparative microglial phenotypes via H3K18 lactylation. Notably, the dual role of MCT1/2 in lactate shuttling and lactylation underscores their therapeutic potential. Engineering blood–brain barrier-penetrant MCT modulators or lactate prodrugs could spatially control lactylation in specific brain regions, mitigating off-target effects.

Beyond neurological pathologies, emerging evidence suggests potential roles of histone lactylation in developmental biology. In embryogenesis, dynamic metabolic shifts—such as the Warburg-like glycolysis prevalent in stem cells—may generate localized lactate pools that could epigenetically regulate gene expression via lactylation. Preliminary studies indicate that lactylation marks (e.g., H3K18la) are enriched in early embryonic tissues and pluripotent stem cells, where they may influence lineage specification by modulating chromatin accessibility at key developmental genes [72]. Further investigation into lactylation’s impact on stem cell self-renewal, differentiation, and tissue morphogenesis could unveil novel mechanisms linking cellular metabolism to developmental programming, with implications for regenerative medicine and organoid models of disease.

By addressing these challenges, future research will refine the mechanistic landscape of histone lactylation and catalyze the development of lactate-centric diagnostics and therapies for neurological disorders.

## Figures and Tables

**Table 1:** Comparative analysis of histone lactylation mechanisms across major neurological disorders.

Disorder	Key Lactylation Sites	Core Pathological Mechanism	Disease Phenotypes
Alzheimer’s disease	H4K12la, H3K18la	PKM2/glycolysis positive feedback loop → microglial hyperactivation	Aβ deposition, cognitive decline, neuroinflammation
Glioblastoma	H3K9la	BRD4 recruitment to enhancers → oncogene activation	Tumor proliferation, therapy resistance, Warburg effect amplification
Ischemic stroke	H3K9la, H3K18la	LDHA-mediated HMGB1 promoter lactylation → NLRP3 inflammasome activation	Infarct volume expansion, blood–brain barrier disruption, neuronal death
Depression	H1Kla	MCT2-mediated neuronal lactate uptake → prefrontal cortex protein lactylation	Neuronal hyperexcitability, behavioral despair, social avoidance
Aging/Neuro-inflammation	H3K18la	Exercise-induced lactate → microglial phenotype switching (pro- to anti-inflammatory)	Cognitive dysfunction, accelerated neurodegeneration, synaptic impairment

## Data Availability

No data were analyzed during the current study.

## Abbreviations

AD	Alzheimer’s disease
AlCl_3_	aluminum chloride
APP	amyloid precursor protein
Arg 1	arginase 1
Aβ	amyloid-β
4-CIN	α-cyano-4-hydroxycinnamate
CNS	central nervous system
D-gal	d-galactose
ECS	electroconvulsive stimulation
ERK	extracellular regulated protein kinases
GLO2	glyoxalase ii
HPLC	high-performance liquid chromatography
BNDF	brain-derived neurotrophic factor
HDAC	histone deacetylase
KAT	lysine acetyltransferase
Kla	lysine lactylation
K_L-la_	l-lactyl-lysine
K_ce_	n-ε-(carboxyethyl)-lysine
K_D-la_	d-lactyl-lysine
LDH	lactate dehydrogenase
LGSH	lactic glutathione
MCT1	monocarboxylate transporter 1
MCT2	monocarboxylate transporter 2
ROS	reactive oxygen species
SIRT	sirtuins
STAT3	signal transducer and activator of transcription 3
OX	oxymate
PKM2	pyruvate kinase m2
PSEN1	presenilin
SASP	senescence-associated secretory phenotype
SAH	subarachnoid hemorrhage
PFC	prefrontal cortex
VEGF	vascular endothelial growth factor
LDHA	lactate dehydrogenase A
BRD4	bromodomain-containing protein 4
